# PREVALENCE AND CHARACTERISTICS OF HEALTH CARE–RELATED TASKS BY INFORMAL DEMENTIA CAREGIVERS: A SYSTEMATIC REVIEW

**DOI:** 10.1093/geroni/igae098.2593

**Published:** 2024-12-31

**Authors:** Mohammed Zuber, Samruddhi Nandkumar Borate, Chia Jie Tan, Niying Li

**Affiliations:** University of Georgia, Athens, Georgia, United States; University of Georgia, Athens, Georgia, United States; University of Utah, Salt Lake City, Utah, United States; University of Georgia, Athens, Georgia, United States

## Abstract

Healthcare-related tasks (e.g. making appointments, talking to providers) performed by informal caregivers caring for older adults with dementia have become increasingly common. This is a systematic review of the prevalence and characteristics of healthcare related tasks performed by informal caregivers. Articles were searched in PubMed, Embase, PsychInfo and CINAHL until September 2023. Studies that were based in the United States and reported the prevalence of healthcare related tasks performed by informal dementia caregivers were included. Tasks identified were grouped into health and medical care, advocacy and care coordination, and surrogate tasks. Caregiver characteristics were reported. Of 6,284 articles screened, we included 12 studies. All the studies were cross-sectional and the majority of them were nationally representative. Majority of caregivers were female and spousal caregivers. Only four studies included racial and ethnic minority caregivers. Medication management, including keeping track of medications, was the most common health and medical care task reported in eight studies ranging from 41% to 86%, followed by five studies reporting 7% to 47% caregivers being involved in wound and skin care. Two studies reported caregivers performing advocacy and care coordination tasks (27 to 73%) such as making appointments, managing insurance, and one study reported surrogate tasks such as participating in medical/treatment decisions (41%). Informal dementia caregivers were involved in a wide range of complex healthcare related tasks. The healthcare system and healthcare professionals need to provide training and support to informal dementia caregivers in performing these tasks.

